# Data describing the eco-physiological responses of twenty-four sunflower genotypes to water deficit

**DOI:** 10.1016/j.dib.2018.10.045

**Published:** 2018-10-18

**Authors:** Nicolas Blanchet, Pierre Casadebaig, Philippe Debaeke, Harold Duruflé, Louise Gody, Florie Gosseau, Nicolas B. Langlade, Pierre Maury

**Affiliations:** a LIPM, Université de Toulouse, INRA, CNRS, Castanet-Tolosan, France; b AGIR, Université de Toulouse, INRA, Castanet-Tolosan, France; c AGIR, Université de Toulouse, INP, ENSAT, Castanet-Tolosan, France

**Keywords:** *Helianthus*, Abiotic stress, Phenotyping platform, Drought stress

## Abstract

This article presents experimental data describing the physiology and morphology of sunflower plants subjected to water deficit. Twenty-four sunflower genotypes were selected to represent genetic diversity within cultivated sunflower and included both inbred lines and their hybrids.

Drought stress was applied to plants in pots at the vegetative stage using the high-throughput phenotyping platform Heliaphen at INRA Toulouse (France). Here, we provide data including specific leaf area, osmotic potential and adjustment, carbon isotope discrimination, leaf transpiration, plant architecture: plant height, leaf number, stem diameter. We also provide leaf areas of individual organs through time and growth rate during the stress period, environmental data such as temperatures, wind and radiation during the experiment. These data differentiate both treatment and the different genotypes and constitute a valuable resource to the community to study adaptation of crops to drought and the physiological basis of heterosis. It is available on the following repository: https://doi.org/10.25794/phenotype/er6lPW7V

**Specifications table**

**Table:** 

Subject area	*Biology*
More specific subject area	*Eco-physiological data.*
Type of data	*Table, .csv files*
How data was acquired	*The Heliaphen robot, an osmometer (Wescor Model 5520, Logan, Utah, USA) and the Stable Isotope Platform SHIVA (University of Toulouse, France)*
Data format	*Processed and filtered data*
Experimental factors	*24 genotypes of Helianthus annuus in two environmental conditions (irrigated or not, fraction of transpirable soil water = 1 or 0.1) with 3 replicates*
Experimental features	*Eco-physiological traits at leaf or plant level and climatologic data.*
Data source location	*The outdoor Heliaphen phenotyping platform at the Institut National de la Recherche Agronomique (INRA) station, Auzeville, France (43 °31’41.8’’N, 1 °29’58.6’’E)*
Data accessibility	*Data are with this article and also publicly available in the SUNRISE Archive depository with following DOI: 10.25794/phenotype/er6lPW7V*
Related research article	H. Badouin, J. Gouzy, C.J. Grassa, F. Murat, S.E. Staton, L. Cottret, C. Lelandais-Brière, G.L. Owens, S. Carrère, B. Mayjonade, L. Legrand, N. Gill, N.C. Kane, J.E. Bowers, S. Hubner, A. Bellec, A. Bérard, H. Bergès, N. Blanchet, M.C. Boniface, D. Brunel, O. Catrice, N. Chaidir, C. Claudel, C. Donnadieu, T. Faraut, G. Fievet, N. Helmstetter, M. King, S.J. Knapp, Z. Lai, M.C. Le Paslier, Y. Lippi, L. Lorenzon, J.R. Mandel, G. Marage, G. Marchand, E. Marquand, E. Bret-Mestries, E. Morien, S. Nambeesan, T. Nguyen, P. Pegot-Espagnet, N. Pouilly, F. Raftis, E. Sallet, T. Schiex, J. Thomas, C. Vandecasteele, D. Varès, F. Vear, S. Vautrin, M. Crespi, B. Mangin, J.M. Burke, J. Salse, S. Muños, P. Vincourt, L.H. Rieseberg, N.B. Langlade, The sunflower genome provides insights into oil metabolism, flowering and Asterid evolution, Nature. 546 (2017) 148–152. doi:10.1038/nature22380. [2]

**Value of the data**

● The data can be used to study drought stress that is an important issue to adapt crops to climate change. Sunflower is particularly impacted as the major oilseed crop in marginal and non-irrigated areas [1]. In this experiment, plants were subjected to two treatments (Well-Watered or Water-Deficit) managed on the outdoor Heliaphen high-throughput phenotyping platform.
● The data can also be used to study heterosis that is the most outstanding phenomenon used by natural selection and mankind to adapt plant and animal organisms to new environments. Twenty-four genotypes of cultivated sunflower comprising four maintainer lines, four restorer lines and their 16 corresponding hybrids were studied and allow studying the physiological basis of heterosis.
● The data comprises 405 eco-physiological traits at leaf or plant level are presented to characterize and quantify the architecture and physiology of sunflowers before and after water-deficit for all genotypes
● The data can be used to study leaf development. Leaf area was measured for individual leaves and dynamically through time to characterize the different leaf expansion and transpiration rates
● All together, these data represent a unique comprehensive description of sunflower responses to drought including a large genetic variability.

## 1 Data

In this data article, we are sharing the eco-physiological responses of 24 genotypes of sunflower grown in two environmental conditions in the outdoor Heliaphen platform. These datasets are part of a larger project that integrate other omics data at different biological levels and be associate to diverse articles.

The data associated with this article can be found at: https://doi.org/10.25794/phenotype/er6lPW7V and the summary of the eco-physiological traits are detailed Table 1.

**Table 1:** Descriptions of the eco-physiological traits.

Column	Description	Unit/Format
PLANT_CODE	Plant ID, used on the Heliaphen platform	
PLANT_NAME	Groups the name of the plant genotype, the treatment applied to the sample and the repetition number	
FTSW	FTSW value the day the plant was sampled	
SLA_MATURE	Specific leaf area in the mature leaf	m^2^/kg
SLA_YOUNG	Specific leaf area in the young leaf	m^2^/kg
OSM_POT_MATURE	Osmotical potential in the mature leaf	MPa
OSM_POT_YOUNG	Osmotical potential in the young leaf	MPa
OSM_POT_100_MATURE	Osmotical potential at full turgor (after rehydration) in the mature leaf	MPa
OSM_POT_100_YOUNG	Osmotical potential at full turgor (after rehydration) in the young leaf	MPa
CID_MATURE	Carbon Isotope Discrimination in the mature leaf	°/_00_
CID_YOUNG	Carbon Isotope Discrimination in the mature leaf	°/_00_
LEAF_EXP_TOT_EST	Leaf expansion during the last 24 h before sampling	mm^2^
LEAF_AREA_TOT_EST	The total leaf area measured the last day	mm^2^
TRANSPIRATION_TOT	Water transpiration per surface unit in the last 24 h	g/mm^2^
LEAF_NB_TOT_START_TREAT	Number of leaf on the plant when the treatment started	
PLANT_HEIGHT_START_TREAT	Plant height when the treatment started	Mm
COLLAR_DIAM_START_TREAT	Collar diameter measured when the treatment started	
LEAF_NB_TOT_RATE	Differential number of leaves counted during a period expressed in degree-days	
PLANT_HEIGHT_RATE	Differential plant height measured during the stress period normalised by the degree-day	mm/dd
COLLAR_DIAM_RATE	Differential collar diameter measured during the stress period normalised by the degree-day	mm/dd

## 2 Experimental design, materials, and methods

### 2.1 Plant material and growth conditions

The experiment was performed from May to July 2013 in the outdoor Heliaphen phenotyping platform at the Institut National de la Recherche Agronomique (INRA) station, Auzeville, France (43 °31’41.8’’N, 1 °29’58.6’’E). This section presents in details the plant material and growth conditions used to produce the data that are highly similar to other experiments performed on the Helipahen platform and described in [2] and [3].

Bleach-sterilized seeds were germinated on Petri dishes with Apron XL and Celeste solutions (Syngenta, Basel, Switzerland) for 78 h at 28 °C. Germinated plantlets were transplanted in individual pots filled with 15 L of P.A.M.2 potting soil (Proveen distributed by Soprimex, Chateaurenard, Bouches-du-Rhône, France) and covered with a 3-mm-thick polystyrene sheet to prevent soil evaporation. After 17 days after germination (DAG), plants were fertilized with 500 ml of Peter׳s Professional 17-07-27 (0,6 g/l) and extra mix composed of oligo-element Hortilon (0,46 g/l) solution. At 21 DAG, Polyaxe at 5 mg/l was applied on foliage against thrips.

In total, 144 plants, corresponding to 24 genotypes (4 males and 4 females and their 16 hybrids obtained by crossing) were grown in two (Well-Watered; WW or Water-Deficit; WD) conditions done in triplicate. Each pot was adequately fertilized and irrigated as in [4] before the beginning of the water deficit application. Pots were saturated with water 35 DAG and after excessive water was drained (~ for two hours), pots were weighed to obtain the full soil water retention mass. Then, irrigation was stopped at 38 DAG (~20-leaf stage corresponding to bud formation phase (stage R1 or R3; [5]) for WD plants (as in work by [3]). All pots were covered with a 3 mm layer of polystyrene sheets at the collar level to limit soil evaporation. Soil evaporation was estimated according to [6]. Both WW and WD plants were weighed three or four times per day by the Heliaphen robot to estimate transpiration [3]. WW plants were re-watered at each weighing by the robot to soil water retention capacity. Pairs of WD and WW plants were harvested when the FTSW of the stressed plant reached 0.1 (occurring between the 42 and the 47 DAG). At harvest, leaves for proteomic analysis were cut without their petiole and immediately frozen in liquid nitrogen from 11 h to 13 h, and two others leaves were cut for physiological traits measurements.

Physiological leaf traits (osmotic potential, carbon isotope discrimination, specific leaf area) were measured on two leaf development stages: mature (LEAF_MATURE) and young (LEAF_YOUNG). Mature leaf developmental stage corresponds to a dark green leaf, assumed to be experiencing its highest photosynthetic rate and having recently reached its maximum size [7]. The young leaf stage corresponded to an expanding green leaf, which lays three nodes above the mature one. The selected leaf to harvest for the proteomic analysis was the *n* + 1 leaves of the mature.

### 2.2 Water deficit experienced by the plant

FTSW (fraction of transpirable soil water) was used as an indicator of water deficit experienced by the plant [8]. The Fraction of Transpirable Soil Water (FTSW) is estimated as FTSW = ATSW/TTSW Where ATSW is the available soil water with ATSW = *w*_*d*_ - (*w*_*full*_ × 0.61). TTSW is the Total Transpirable Soil Water, with TTSW = *w_full_* (1 – 0.39); *w*_*full*_ is the weight of the pot at soil water retention capacity, *w*_*d*_ is the weight of th pot on day *d*.

### 2.3 Leaf area and expansion

The width (*w*) and length (*l*) of each leaf were measured every two days. The leaf area (LEAF_AREA, *a*) was determined as: *a* = 0:7 ×*w* x *l*. The expansion (LEAF_EXP, *Δa*) was determined as: *Δa* = *a*^*n*^ - *a*^*n-1*^ where *a*^*n*^ is the leaf area at day *n*.

The specific leaf area (SLA) was determined with discs (3.1 cm diameter) cut on rehydrated lamina of sampled leaves and dried (48 h, 80 °C). SLA was calculated as leaf area/leaf dry weight (m^2^.kg^-1^) as described in [9].

### 2.4 Osmotic potential

Both leaf osmotic potential (OSM_POT) and osmotic potential at full turgor (OSM_POT_100) were measured on expressed sap of frozen and thawed leaves using 10 ml aliquots placed in an osmometer (Wescor Model 5520, Logan, Utah, USA) calibrated with manufacturer solutions, as described in [10]. Briefly, osmotic potential at full turgor (OSM_POT_100) was determined after 24 h leaf rehydration at 4 °C in a dark room by placing the petioles in a container with distilled water. Osmotic potentials were determined by converting the osmometer reading (mmol kg^-1^), using the van׳t Hof relation (OSM_POT = -RTdc), where R is the gas constant, T is the temperature in Kelvin, d is the density of water at temperature T and c is the concentration of osmotically-active solutes, given by the osmometer.

### 2.5 Carbon isotope discrimination

Carbon isotope discrimination (CID) refers to the ratio of the carbon isotopes 13 C/12 C in plant material, relative to the same ratio in the atmosphere. In order to assess CID, the same samples of oven-dried leaves used for SLA measurements were ground. Subsample of 3 mg was weighed and placed in capsules (Elemental Microanalysis, Okehampton, UK) and analysed using a continuous low isotope ratio MS at Stable Isotope Platform SHIVA (University of Toulouse, France). Carbon isotope composition was calculated relative to the international Pee Dee Belemnite standard and CID was estimated according to [11].

### 2.6 Daily plant transpiration

The plant transpiration rate was defined as the amount of water lost per leaf surface unit in a given interval of time (24 H). The amount of water lost (*t*^*tot*^) is estimated by computing the difference in pot weight (*w*^*n*^_*water*_) on day n and pot weight before watering (*w*^*n+1*^_*dry*_) on day *n*+1. *t*^*tot*^ = *w*^*n*^_*water*_ - *w*^*n+1*^_*dry*_. To take into account the amount of plant leaf area through which the water is lost, *t*^*tot*^ is then divided by the mean plant leaf area between the two days. Where *tf* [*tf* = *t*^*tot*^/((*a*^*n*^_*tot*_+*a*^*n-1*^_*tot*_)/2)] is the whole plant transpiration rate (TRANSPIRATION_TOT), *a*^*n*^_*tot*_ is the total leaf area of the plant at day *n*.

### 2.7 Plant trait measurements

Collar diameter (COLLAR_DIAM) and the plant height (PLANT_HEIGHT) were measured and the number of leaves (LEAF_NB) counted at the beginning (_START_TREAT) and at the end (_END_TREAT) of the stress period. Finally, the differential was calculated between these two dates and normalised using the degree-day of this period (_RATE).

The leaf area of the entire plant (LEAF_AREA_TOT) and the leaf expansion (LEAF_EXP_TOT) was recorded at the same date and estimates for the stress period.

### 2.8 Climatic data

Climatic metadata of the agrometeorological database of INRA collected at the Auzeville-Tolosane – Station N°31035002– (43 °31׳44.4"N, 1 °30׳14.4"E).

### 2.9 Data curation and organization

The aberrant values deleted of the trait measurements were determined as plus or minus three times the standard deviation. They were removed from the dataset.

The data is available as a series of csv, xls and pdf files with the SUNRISE_13HP02_ prefix. Comma-separated values files are automatically generated by the information system “SUNRISE Phenotype Archive” from xls (tab-delimited files) and are equivalent.

Four files include physiological data:

– …Physio_Correspondance.csv with start and end of treatments and leaf ranks of sampled leaves
– …Physio_classe_with_threshold.csv with all physiological data at the plant and leaf levels at the start and/or end of the treatment as detailed in Table 1
– …LIPM_daily_plant.csv with all temporal data measured during the experiment at the plant level
– …LIPM_leaves_daily.csv with all temporal data measured during the experiment at the individual leaf level

The file …LIPM_METEO.csv describes the climatic data during the treatment period. The file …LIPM_GENOTYPE.csv describes the pedigree of the genotypes and the seed stocks used for the measured plants and their parents in case of hybrids. The files …LIPM_ITK.pdf and …LIPM_MAP.pdf describe the plant technical management (fertilization and insecticide treatments) and the plant positions on the Heliaphen phenotyping platform.

Finally, SUNRISE_METADATA_V5.csv provides the code and natural description of studied variables together with their informatics format for a facilitated re-use of these data.
