# Supplementary material for: Data describing the eco-physiological responses of twenty-four sunflower genotypes to water deficit
Source: Data Brief. 2018 Oct 18;21:1296–301. doi: 10.1016/j.dib.2018.10.045 (PMC6231244; doi:10.1016/j.dib.2018.10.045)
Supplement: Supplementary file 1 — Transparency document [file mmc1.pdf]

CONFLICT OF INTEREST STATEMENT

AUTHOR DECLARATION

We wish to confirm that there are no known conflicts of interest associated with this publication and there has been no significant financial support for this work that could have influenced its outcome.

We confirm that the manuscript has been read and approved by all named authors and that there are no other persons who satisfied the criteria for authorship but are not listed. We further confirm that the order of authors listed in the manuscript has been approved by all of us.

We confirm that we have given due consideration to the protection of intellectual property associated with this work and that there are no impediments to publication, including the timing of publication, with respect to intellectual property. In so doing we confirm that we have followed the regulations of our institutions concerning intellectual property.

We understand that the Corresponding Author is the sole contact for the Editorial process (including Editorial Manager and direct communications with the office). He is responsible for communicating with the other authors about progress, submissions of revisions and final approval of proofs. We confirm that we have provided a current, correct email address which is accessible by the Corresponding Author and which has been configured to accept email from nicolas.langlade@inra.fr

Signed by all authors as follows:

Nicolas Blanchet<br>*1 October 2018*	
Pierre Casadebaig<br>*1 October 2018*	
Philippe Debaeke<br>*1 October 2018*	
Harold Duruflé<br>*1 October 2018*	
Louise Gody<br>*1 October 2018*	
Florie Gosseau<br>*1 October 2018*	
Nicolas B. Langlade<br>*1 October 2018*	
Pierre Maury<br>*1 October 2018*	
